# Effects of smoke-free air law on acute myocardial infarction hospitalization in Indianapolis and Marion County, Indiana

**DOI:** 10.1186/s12889-018-5153-y

**Published:** 2018-02-09

**Authors:** Anne M. Weaver, Yi Wang, Katelin Rupp, Dennis P. Watson

**Affiliations:** 1 Department of Environmental Health Sciences, Indiana University Richard M. Fairbanks School of Public Health, 1050 Wishard Blvd, Indianapolis, IN 46202 USA; 2 Indiana State Department of Health, Tobacco Prevention and Cessation Commission, Indianapolis, IN USA; 3 Department of Social and Behavioral Sciences, Indiana University Richard M. Fairbanks School of Public Health, Indianapolis, IN USA

**Keywords:** Tobacco cessation, Smoking ban, Myocardial infarction, Hospital admission, Tobacco policy

## Abstract

**Background:**

A comprehensive smoke-free air law was enacted on June 1, 2012 in most of Marion County, Indiana, including all of the City of Indianapolis. We evaluated changes in acute myocardial infarction (AMI) admission rates in Indianapolis and Marion County before compared to after the law.

**Methods:**

We collected AMI admissions from five Marion County hospitals between May 2007 and December 2014. We used Poisson regression to evaluate the overall effects of the law on monthly AMI hospitalizations, adjusting for month, seasonality, meteorology, air pollution, and hospital utilization. We tested the interactions between the law and AMI risk factors on monthly AMI admission rates to identify subpopulations for which the effects might be stronger.

**Results:**

Monthly AMI admissions declined 20% (95% CI 14–25%) in Marion County and 25% (95% CI 20–29%) in Indianapolis after the law was implemented. We observed decreases among never (21%, 95% CI 13–29%), former (28%, 95% CI 21–34%), and current smokers (26%, 95% CI 11–38%); Medicaid beneficiaries (19%, 95% CI 9–29%) and non-beneficiaries (26%, 95% CI 20–31%). We observed decreases among those with a history of diabetes (Yes: 22%, 95% CI 14–29%; No: 25%, 95% CI 18–31%), congestive heart failure (Yes: 23%, 95% CI 16–30%; No: 24%, 95% CI 17–31%), and hypertension (Yes: 23%, 95% CI 17–28%: No: 26%, 95% CI 15–36%).

**Conclusions:**

We observed decreases in AMI admissions comparable with previous studies. We identified subpopulations who benefitted from the law, such as former and current smokers, and those without comorbidities such as congestive heart failure and hypertension.

**Electronic supplementary material:**

The online version of this article (10.1186/s12889-018-5153-y) contains supplementary material, which is available to authorized users.

## Background

Coronary heart disease due to tobacco smoking and secondhand tobacco smoke exposure contributed to approximately 99,300 deaths annually in the United States, as of 2009 [1]. Coronary heart disease contributes to acute myocardial infarctions (AMI) [1, 2]. A number of previous studies have reported a decrease in incidence of AMI after smoke-free legislation implementation in the United States, Europe, and Canada [3–15]. The majority of these studies showed a varying (5–70%), but statistically significant, decline in hospital admissions from AMI in their respected geographical locations. However, it is unknown whether specific subgroups benefit more from smoke-free air laws compared to others.

Marion County, Indiana is the most populous county and is located in central Indiana. The city of Indianapolis is its population center. In 2006, the city of Indianapolis implemented an ordinance prohibiting smoking in most restaurants and workplaces [16]. This law covers most of Marion County, Indiana, with the exception of the cities of Beech Grove, Lawrence, and Southport, and the Town of Speedway. The Town of Speedway issued a less comprehensive law that exempts bars in 2006. The Indianapolis ordinance was amended on June 1, 2012 to include all businesses, most notably prohibiting smoking in bars and taverns, with exemptions including tobacco-specialty businesses and off-track betting facilities [16]. The 2012 amendment also prohibited the use of electronic cigarettes (e-cigarettes) in all locations where smoking was prohibited. On July 1, 2012 the State of Indiana implemented its smoke-free air law, which prohibited smoking in restaurants, most indoor public places, and within eight feet of public entrances [17]. The state law allowed for local ordinances to have precedence over the state if the local ordinance is more strict, which was the case in the city of Indianapolis [16, 17]. The city ban included all of the aspects mentioned in the state ban, but additionally included bars, taverns, and e-cigarettes. The City of Lawrence enacted a smoke-free air law, similar to the Indianapolis law, on October 1, 2012.

Our objective for this study was to evaluate the intermediate and long-term health effects of the 2012 amendment to the Indianapolis smoke-free air law. Therefore, we have evaluated the law’s effects on AMI hospital admissions in the general population of Marion County as well as among specific sub-groups, defined by sex, race, smoking status, Medicaid status and comorbidities (diabetes, hypertension, congestive heart failure).

## Methods

We conducted a retrospective hospital records review study, reviewing medical records before compared to after the smoking ban among five major hospitals in Marion County, Indiana. We obtained data on AMI hospital admissions and emergency department (ED) visits for five major hospitals in Marion County from electronic medical records of the Indiana Network of Patient Care (INPC) at the Regenstrief Institute. Admissions and ED visits in these hospitals account for > 95% of all hospitalizations in Marion County. We obtained ED visits and hospital admissions for primary and secondary diagnoses of AMI that occurred from May 1, 2007 through December 31, 2014. We used International Classification of Diseases, 9th version (ICD-9) code 410 or ICD-10 code I21 or I22 to define primary or secondary diagnosis of AMI. We also included information on preexisting conditions that may increase risk of AMI, including diabetes, congestive heart failure (CHF), or hypertension in order to assess if these patients would benefit more or less from the smoke-free air law. If a patient was admitted more than once for AMI during the study period, we included all AMI admissions and ED visits at least 28 days apart. In this report, we referred to all hospital admissions and ED visits in which AMI was a primary or secondary diagnosis as AMI admissions.

We determined the geographical location of each AMI patient’s residential addresses at a zip code level. We classified AMI admissions by zip codes within Indianapolis city limits and those within Marion County; for this analysis, we excluded patients who resided outside of Marion County. We considered Indianapolis and Marion County separately, as there were some locations (Speedway, Beech Grove, and Southport) in Marion County with less strict smoke-free airs compared to Indianapolis. We determined each patient’s smoking status (never smoked, current smoker, or former smoker) via Current Procedural Terminology codes. We stratified by smoking status to determine relative benefit of the smoke-free air law by smoking status. Patients were defined as a current or former smoker if they ever answered yes to the question “Are you a smoker?” or were diagnosed with a tobacco use disorder, toxic effect of tobacco, or history of tobacco use. Former smokers were identified as smokers in the past but have more recently identified as non-smokers using the same criteria. Smoking status was determined based on the encounter most recent to the date of admission. We obtained information on whether each patient had been a Medicaid beneficiary and if the patient had any insurance coverage based on his/her latest insurance information at the time of admission. We also obtained demographic information including, age at admission, sex, and race. We were unable to individually contact patients; as such, all demographic information is gathered from medical records.

We obtained annual total population counts of Marion County by sex and race for each year 2007–2012 from the US Census, as well as projections for 2013 and 2014 (Woods & Poole Economics, Inc. Washington, D.C. Copyright 2015) (Additional file 1: Tables S1 and S2). We obtained age-specific population estimates for the entirety of Marion County for each year 2007–2014 from the US Census, based on age group: < 65, 65–74, and ≥75 years (census.gov). We chose to use these age cutpoints to reflect both changing social behaviors around the age of retirement (commonly 65) as well as increasing risk of AMI with age. To account for the trend of monthly hospital uses in Marion County, we obtained monthly all-cause hospital discharges in Marion County by sex and race for May 2007–December 2013 from the Marion County Health Department and for January–December 2014 from the Indiana State Department of Health.

We obtained hourly temperature and dew point temperatures from 2007 to 2014 for all Indiana stations from the National Oceanic and Atmosphere Administration’s Integrated Surface Database. To account for the effects of temperature variations on AMI admission, we calculated apparent temperatures for each zip code using the formula (apparent temperature = − 2.653 + [0.994 × 24-h mean air temperature (°C)] + [0.0153 × 24-h mean dew point temperature (°C)^2^]), as described in Wilker et al. [18]. To account for the effects of ambient air pollution on AMI admission [19], hourly fine particulate matter (PM_2.5_) levels were downloaded from the U.S. Environmental Protection Agency’s website, and were averaged for each day. Daily PM_2.5_ levels were averaged at a zip code level using inverse distance weighted method based on data from stations within 200 km of a zip code centroid, and where there were at least 18 h of valid readings. We then estimated each patient’s average monthly ambient PM_2.5_ and apparent temperature exposure based on their zip code of residence.

In descriptive tables, we show the mean monthly incidence of AMI hospitalizations in Marion County before and after the smoke-free air law by: sex, age (< 65, 65–74, ≥75), race (black, white, other/unknown), smoking status (never, former, current), whether or not the patient was a Medicaid beneficiary, diabetes status, CHF status, and hypertension status. We also graphed quarterly AMI admissions (per 100,000 residents) from May 1, 2007 to December 31, 2014 in Marion County for black males and females and white males and females by age group. We chose to graph AMI admissions by quarter (three-month intervals) in order to improve clarity of the graphics. All analyses are approved by Indiana University Institutional Review Board.

### Regression analyses

Given that sex and race have been shown to be important risk factors for AMI [20], we used Poisson regression to determine whether the monthly AMI admission rates differed in the population by sex and race. Without inclusion of the smoke-free air law, we modeled monthly AMI admission counts using Poisson regression adjusting our models for month (time trend), seasonality (annual sine and cosine terms) [21], monthly mean apparent temperature (at zip-code level), monthly ambient PM_2.5_ concentrations (at zip-code level), and total monthly all-cause hospital discharges (by sex and/or race) in Marion County. We modeled sex-specific monthly AMI admission rates by using natural logarithms of estimated sex- and age-specific population of Marion County as a model offset when modeling sex; natural logarithms of race- and age-specific population counts as an offset when modeling race; and natural logarithms of sex-, race- and age-specific population counts as an offset when modeling sex and race. All analyses of race were limited to black and white individuals, due to few patients who self-identified as other races and others with missing information on race.

To determine percent change in the monthly AMI admission rate after the smoke-free air law (compared to before the law), we used Poisson regression to model monthly AMI admission rates (counts divided by person-years) with natural logarithm of age-specific Marion County population as an offset. Poisson regression is commonly used in studies examining differences in AMI admission rates in response to smoking bans [4–8], allowing comparability of our study to others. We conducted analyses among all residents of Marion County and stratified by risk factors including sex, race, sex and race, smoking status, Medicaid recipient status, and history of diabetes, congestive heart failure (CHF), and hypertension. All models were adjusted for time trend (linear term), seasonality (annual sine and cosine terms) [21], monthly mean apparent temperature (at zip-code level), monthly total hospital discharges, and monthly ambient PM_2.5_ concentrations (at zip-code level). To examine whether there might be subpopulations in which the effects of smoke-free air law are stronger, we tested the potential interaction between smoke-free air law and these risk factors by adding interaction terms to each model. As sensitivity analyses, we limited our analysis to residents in Indianapolis and to those with a primary diagnosis of AMI (excluding those with AMI secondary to another condition). Analyses were conducted using SAS version 9.4 (SAS Institute, Inc., Cary, NC). A two-sided *p* value of < 0.05 was considered statistically significant.

## Results

### Descriptive results

Table 1 shows the mean age-adjusted monthly incidence of AMI admissions in Marion County before compared to after the smoke-free air law, overall as well as by demographics (age group, sex, and race), smoking status, Medicaid status, history of preexisting conditions (diabetes, congestive heart failure, hypertension), primary diagnosis of AMI, and address within the city of Indianapolis. Overall, admissions of AMI decreased from a mean of 62.3 per 100,000 per month prior to the ban to 51.5 per 100,000 per month after the ban. Those aged 75 years and over had the highest age-specific incidence of AMI both before and after the ban (105.3 and 90.7 per 100,000, respectively); incidence of AMI decreased among those 65–74 and 75 years and older. Incidence decreased in males, current smokers, those of other/unknown race, those who were not Medicaid beneficiaries, those who were not diabetic, those with primary diagnosis of AMI, and those who lived within Indianapolis city limits. Relatively few cases occurred in persons of other/unknown race. Incidence of AMI decreased regardless of CHF or hypertension status, although incidence was higher among those with CHF and hypertension status.

**Table 1:** Monthly age-adjusted incidence of AMI admissions per 100,000 residents of Marion County before and after smoking ban

	Pre-ban	Post-ban
	(5/1/2007–5/31/2012)	(6/1/2012–12/31/2014)
	Mean (SD)	Mean (SD)
Total AMI admissions*	62.3 (41.5)	51.5 (36.1)
Age		
< 65	10.7 (1.7)	10.2 (1.5)
65–74*	71.0 (14.2)	53.5 (14.5)
≥75*	105.3 (18.7)	90.7 (20.5)
Sex		
Female	32.3 (25.1)	27.2 (21.7)
Male*	30.0 (18.6)	24.2 (16.3)
Smoking status		
Never	25.9 (20.7)	24.1 (20.0)
Former	27.0 (17.9)	23.7 (16.2)
Current*	9.4 (7.5)	4.1 (3.8)
Race		
Black	15.2 (11.3)	14.3 (10.7)
White	37.4 (25.2)	33.2 (23.9)
Other/unknown*	9.7 (8.4)	4.2 (4.3)
Medicaid beneficiary		
Yes	12.5 (8.9)	12.1 (8.0)
No*	49.8 (34.9)	39.4 (29.3)
Diabetes		
Yes	27.5 (18.6)	23.7 (16.9)
No*	34.8 (25.1)	27.8 (20.3)
CHF		
Yes*	36.7 (27.3)	26.9 (22.8)
No*	25.6 (16.1)	24.5 (16.0)
Hypertension		
Yes*	50.6 (34.1)	42.1 (30.5)
No*	11.8 (9.4)	9.4 (7.2)
Primary diagnosis of AMI*	38.4 (24.5)	29.5 (19.2)
Indianapolis only*	59.3 (39.4)	48.1 (33.6)

*p < 0.05 (t-test)

**Fig. 1:**
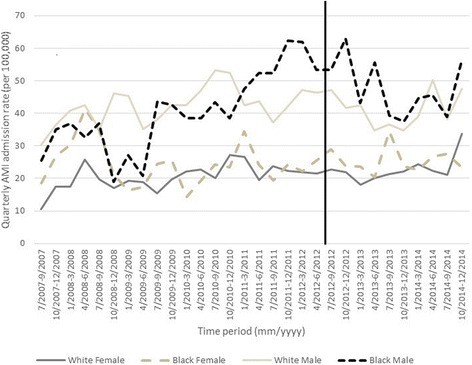
Quarterly AMI admission rate per 100,000 by sex and race for residents of Marion County less than 65 years old. Vertical line indicates ordinance date

**Fig. 2:**
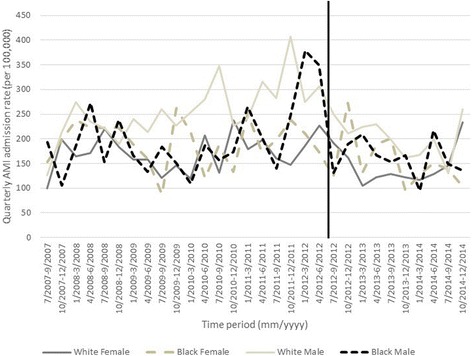
Quarterly AMI admission rate per 100,000 by sex and race for residents of Marion County less than 65–74 years old. Vertical line indicates ordinance date

**Fig. 3:**
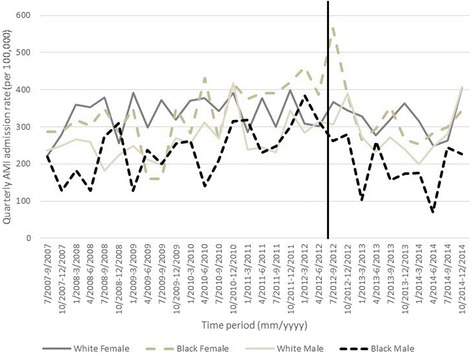
Quarterly AMI admission rate per 100,000 by sex and race for residents of Marion County 75 years and older. Vertical line indicates ordinance date

We graphed quarterly AMI admission rates (per 100,000 residents) from July 1, 2007 to December 31, 2014 in Marion County by sex and race, shown separately for each age group: < 65, 65–74, ≥75 years old (Figs. 1, 2 and 3). Among those < 65, white and black females had consistently lower AMI rates compared to white and black males; overall admission rates were similar by race with sexes. Among those 65–74, AMI admission rates were similar in strata of age and race, but white males appeared to have the overall highest incidence. Among those ≥75 year, females appeared to have a generally higher AMI admission rate compared to males, although rates were similar by race and sex; black females appeared to have the highest rates. Overall, we did not observe a clear decrease in AMI rates after the smoke-free air law.

We conducted Poisson regression models (without smoke-free air law) examining sex and race as potential risk factors for AMI admission in our population (Table 2). We concluded that sex and race are potential risk factors for AMI admissions in our population. Males had a 73% (95% CI 44%, 107%) higher monthly AMI admission rate compared to females. Compare to white individuals, monthly AMI admission rates for black individuals were higher by 13% (95% CI 2%, 26%). Compared to white females, monthly AMI admission rates for black females, white males, and black males were higher by 35% (95% CI 19%, 54%), 66% (95% CI 52%, 82%) and 81% (95% CI 53%, 114%), respectively. These differences were all statistically significant.

**Table 2:** Estimated percent differences in average monthly incidence rate of AMI admissions from Poisson regression in Marion County, by sex, race, and sex and race^a, b^

	Number (%) of admissions	% difference in incidence rate (95% CI)
Sex		
Females	6707 (44.4%)	REF
Males	8397 (55.6%)	73% (44%, 107%)
Race		
White	9209 (61.0%)	REF
Black	4047 (26.8%)	13% (2%, 26%)
Sex and Race		
White Females	3905 (25.9%)	REF
Black Females	1967 (13.0%)	35% (19%, 54%)
White Males	5304 (35.1%)	66% (52%, 82%)
zBlack Males	2080 (13.8%)	81% (53%, 114%)

^a^Adjusted for month (time trend), seasonality (annual sine and cosine terms), monthly mean apparent temperature (at zip-code level), monthly ambient PM2.5 concentrations (at zip-code level), and total monthly hospital discharges. Offset was the log of the total age- sex- and/or race- specific population of Marion County

^b^Missing/excluded: race—131 (0.87%) Hispanic or Latino, 12 (0.08%) American Indian/Alaska Native, 27 (0.18%) Asian, 404 (2.67%) Native Hawaiian/Pacific Islander, 29 (0.2%) multiracial, 261 (1.73%) other race, 984 (6.51%) unknown/missing race

We conducted stratified Poisson regression of percent changes in monthly AMI admission rates after the smoke-free air law (Table 3) compared to before. We observed these associations among those who lived anywhere in Marion County (20% decrease, 95% CI 14%, 25%) as well as those living in Indianapolis (25% decrease, 95% CI 20%, 29%). We observed statically significant decreases in monthly AMI admission rates after the smoke-free air law went into effect for all subgroups except black females. White females had a 22% (95% CI 12%, 31%) decrease, white males had a 19% (95% CI 11%, 27%) decrease, and black males had a 21% (95% CI 7%, 33%) decrease in AMI admission rate. By sex, there was a 26% (95% CI 20%, 32%) decrease in monthly AMI admission rate among males and a 20% (95% CI 12%, 27%) decrease among females. Black individuals had a 15% (95% CI 4%, 25%) decrease and white individuals had a 21% (95% 15%, 27%) decrease in AMI admissions.

**Table 3:** Estimated percent change in monthly incidence rate after the smoking ban (95% CI) from Poisson regression in Marion County and Indianapolis, and in strata of risk factors (sex, race, smoking status, Medicaid, and diabetes, congestive heart failure, and hypertension)^a, b^

	Number (%) of admissions	% change in incidence rate (95% CI)	*P*-value for interaction ^c^
Location			
Marion County	15,104 (100%)	-20% (− 25%, − 14%)	
Indianapolis only	14,392 (95.3%)	−25% (− 29%, − 20%)	
Sex			0.53
Males	8397 (55.6%)	−26% (−32%, − 20%)	
Females	6707 (44.4%)	−20% (− 27%, − 12%)	
Race^b^			0.15
Black	4047 (26.8%)	−15% (− 25%, − 4%)	
White	9209 (61.0%)	−21% (− 27%, − 15%)	
Sex and Race^b^			
White Females	3905 (25.9%)	− 22% (− 31%, − 12%)	REF
Black Females	1967 (13.0%)	−2% (− 17%, 17%)	< 0.0001
White Males	5304 (35.1%)	−19% (−27%, −11%)	< 0.0001
Black Males	2080 (13.8%)	− 21% (−33%, −7%)	< 0.0001
Smoking			
Never	5530 (36.6%)	− 21% (− 29%, −13%)	REF
Former	7421 (49.1%)	−28% (−34%, − 21%)	0.03
Current	2153 (14.3%)	− 26% (−38%, − 11%)	< 0.0001
Medicaid beneficiary			< 0.0001
Yes	3902 (25.8%)	− 19% (− 29%, − 9)	
No	11,202 (74.2%)	−26% (− 31%, − 20%)	
History of Diabetes			0.41
Yes	6491 (43.0%)	−22% (− 29%, − 14%)	
No	8613 (57.0%)	−25% (− 31%, − 18%)	
History of CHF			< 0.0001
Yes	7308 (48.4%)	−23% (− 30%, − 16%)	
No	7796 (51.6%)	−24% (− 31%, − 17%)	
History of Hypertension			0.03
Yes	11,933 (79.0%)	− 23% (− 28%, − 17%)	
No	3171 (21.0%)	− 26% (− 36%, − 15%)	
Limited to primary AMI	9956 (65.9%)	−16% (− 23%, − 9%)	

^a^Adjusted for month (time trend), seasonality (annual sine and cosine terms), monthly mean apparent temperature (at zip-code level), monthly ambient PM2.5 concentrations (at zip-code level), and total monthly hospital discharges. Offset was the log of the total age-specific population of Marion County

^b^Missing/excluded: race—131 (0.87%) Hispanic or Latino, 12 (0.08%) American Indian/Alaska Native, 27 (0.18%) Asian, 404 (2.67%) Native Hawaiian/Pacific Islander, 29 (0.2%) multiracial, 261 (1.73%) other race, 984 (6.51%) unknown/missing race

^c^Interaction between smoking ban and these risk factors assessed by adding interaction terms to each model

Additionally, there was a 21% (95% CI 13%, 29%) decrease among never smokers, a 28% (95% CI 21%, 34%) decrease among former smokers, and a 26% (95% CI 11%, 38%) decrease among current smokers. We observed a 19% (95% CI 9%, 29%) decrease among Medicaid beneficiaries and a 26% (95% CI 20%, 31%) decrease among those who have never had Medicaid. We observed decreases in monthly AMI admission rates among those with (22%, 95% CI 14%, 29%) and without (25%, 95% CI 18%, 31%) a history of diabetes, those with (23%, 95% CI 16%, 30%) and without (24%, 95% CI 17%, 31%) a history of CHF, and among those with (23%, 95% CI 17%, 28%) and without (26%, 95% CI 15%, 36%) a history of hypertension. These associations were also observed when limiting to those with a primary diagnosis of AMI (16%, 95% CI 9%, 23%). We observed statistically significant interactions between smoke-free air law status and race/sex categories, smoking status, Medicaid beneficiary status, history of CHF, and history of hypertension.

## Discussion

The Indianapolis smoke-free air law was a public health ordinance designed to eliminate individuals’ exposure to tobacco smoking and secondhand smoke in indoor public places and places of work. We evaluated the effect of the smoke-free air law, effective June 1, 2012, on AMI admissions in Marion County. We found a 20% decrease in the monthly AMI admission rate after the ban among residents in all of Marion County, as well as a 25% decrease among those in Indianapolis specifically. As Indianapolis residents are subject to the smoke-free air law, but other residents of Marion County may not be, this result aligns with our expectations of seeing stronger associations where the ban was in effect. We examined changes in AMI rates by sex, race, sex/race strata, smoking status, Medicaid status, diabetes history, CHF history, hypertension history, and limited to a primary diagnosis of AMI. All groups except black females had decreases in AMI admissions after the ban. In addition, we identified potential interactions, indicating that the effect of smoke-free air law on AMI admissions may be different by sex/race category, smoking status, Medicaid status, CHF status, and hypertension status. These results are evidence of the public health benefits provided to the population of Marion County from the smoke-free air law. The overall decreases in admissions were consistent with other studies [3, 7, 8].

Our study examined sex and race as risk factors for AMI in more depth than other studies that examined the effects of smoke-free air laws on AMI. In regression analyses, we found that males had a 26% decrease in monthly AMI admission rates, females had a 20% decrease, black individuals had a 15% decrease, and white individuals had a 21% decrease. White females had a 22% decrease, white males had a 19% decrease, and black males had a 21% decrease. Interaction analyses indicated that the effect of the smoke-free air law on monthly AMI admission rates was different by sex/race group. Only black females did not appear to benefit by the smoke-free air law. Similar to our results, Cesaroni et al. observed a stronger effect of a smoking ban on risk of AMI among males [7]. We are not aware of other studies that examined these associations by race.

We observed a decrease in monthly AMI admission rates among current smokers (26%), former (28%) and never smokers (21%). The interaction between smoke-free air law and smoking status was significant, indicating current smokers and former have a greater decline in monthly AMI admission rates compared to never smokers. The results of this study, which showed the greatest decline among current smokers, contradicted the literature regarding the relationship between smoking status and AMI. The literature suggested a decrease in AMI among smokers, but the greatest decrease was observed in individuals identifying themselves as “never smokers” or “non-smokers” [6, 22]. It is possible that we observed a decrease in monthly AMI admissions among current smokers if smokers living under a smoke-free air law smoked fewer cigarettes, and, therefore, were at a lower risk of AMI. Studies in Italy and Canada have shown decreased cigarette consumption and reduction in the number of smokers after implementation of smoke-free air laws [7, 12]. The Behavioral Risk Factor Surveillance System (BRFSS) reported a decrease in the prevalence of smoking in Indiana from 25.6% in 2011 to 22.9% in 2014 [23]. Therefore, the reduction in monthly AMI admission rates observed in current smokers was at least in part attributable to the decreased prevalence of smoking in Indiana.

We observed a 19% reduction in monthly AMI admission rates among those who were Medicaid beneficiaries, a proxy measure of socioeconomic status, and a 26% decrease among those who were not Medicaid beneficiaries. The interaction term between smoke-free air law and Medicaid status was significant, indicating a greater decrease in monthly AMI admission rates among those who were of higher socioeconomic status (not Medicaid beneficiaries) compared to those of lower socioeconomic status (Medicaid beneficiaries). In contrast, Cesaroni et al. found that some of the largest decreases in their Italian study population were among those who lived in low socioeconomic tracts [7]. Medicaid status might not be a good surrogate of socioeconomic status in our study due to potential misclassification of data obtained from electronic medical records. Our study is the first in the US to attempt to identify subpopulations whose AMI admission rates associated with a smoke-free air law might by Medicaid status.

When evaluating change in monthly AMI admission rates among those with and without comorbidities, we found statistically significant decreases of 20% or more among those with and without diabetes, CHF, and hypertension. These decreases were greater among those without comorbidities, but the interaction term was not statistically significant for diabetes. Our results showed significant decreases for monthly AMI admission rates among individuals with and without hypertension; a greater decrease was observed among those without hypertension. Hypertension is common, occurring in approximately one-third of the American population and is extremely common (76%) among AMI patients in our study; a decrease in AMI admissions among those with hypertension could have great public health importance [21]. The results of this study suggested that individuals without preexisting risk factors for AMI (diabetes, CHF, hypertension) benefited the most from the smoking ordinance. It is possible that preexisting medical conditions may limit some individuals’ social activities and, therefore, they may not be in bars and restaurants and thus would not be affected by the smoke-free air law. Other studies on smoke-free air laws and AMI incidence did not present results stratified by diabetes, CHF, or hypertension status. Studies that examined secondhand smoke exposure as a risk factor of AMI had differing results by diabetes status [24, 25]. These studies show that the risk of AMI among those exposed to secondhand smoke did not differ by hypertension status [24, 25]. It is unclear whether those with preexisting risk factors for AMI would see an additional benefit from a smoking ban.

This study had limitations. First, although we had detailed information on AMI patients, we did not have detailed information on risk factors of AMI in the general population. Therefore, we were unable to determine whether AMI admission rates decreased among individuals with comorbid conditions or if the prevalence of the comorbid conditions decreased. We had no information on other common risk factors of AMI, such as obesity, nutrition, or physical activity. Second, it is possible that temporal trends of smoking prevalence may have affected our estimates. For instance, if smoking prevalence decreased over time, we would have seen a decrease in AMI admission rates among smokers that may not be due to the ban. Third, there was potential misclassification on self-reported factors, such as race and smoking status in our study. Approximately 8.4% of AMI patients had missing or unknown race, but those who had missing or unknown race were excluded from analyses for race. Additionally, due to small numbers, we did not examine AMI admission rates among those who were neither black nor white (approximately 4% of admissions). Fourth, this study relied on electronic medical records for patient information. Therefore, we were unable to include certain potentially relevant factors, such as occupation, diet, dyslipidemia, or activities outside of Marion County.

This study also had strengths. First, we conducted thorough analyses, including stratification by known risk factors for AMI (sex, race, smoking status, Medicaid status, history of diabetes, CHF, hypertension) and interaction between smoke-free air law and these risk factors. Similar studies offered minimal examination of AMI rates in strata of risk factors nor did they check for interaction in as much depth as we did in this report. Second, our regression models accounted for month of admission, seasonality, apparent temperature, ambient PM_2.5_ concentrations, and monthly hospital utilization. Third, our data included > 95% of AMI admissions in Marion County, allowing us confidence that our data are representative of AMI in Marion County.

Overall, we observed a 20% decrease in AMI admissions in the period of six months after the smoke-free air law in Marion County. Our study showed great benefit for all sex/race groups except black females in Marion County. In conclusion, our results were consistent with other studies that demonstrated reduced AMI risk after enforcement of a smoke-free air law.

## Conclusions

AMI admission rates declined in Indianapolis and Marion County in the eighteen months following the implementation of the comprehensive smoke-free air law. This decline was particularly notable among black males and current smokers. The decrease in AMI admission rates was also significant among former smokers, those who were not Medicaid beneficiaries, those without a history of diabetes, those without a history of congestive heart failure, and those with and without a history of hypertension. Our results showed that the Indianapolis comprehensive smoke-free air law is associated with decreased rate of AMI admissions in Marion County. Similar smoking laws may be effective at preventing AMIs in other locations.

## Additional file

Additional file 1: Supplementary Materials. **Table S1.** Marion County population by sex and age. **Table S2.** Marion County population by race and age. (PDF 43 kb)

## Abbreviations

AMI: Acute myocardial infarction
BRFSS: Behavioral Risk Factor Surveillance System
CHF: Congestive heart failure
ED: Emergency department
