# Supplementary material for: Effects of smoke-free air law on acute myocardial infarction hospitalization in Indianapolis and Marion County, Indiana
Source: BMC Public Health. 2018 Feb 9;18:232. doi: 10.1186/s12889-018-5153-y (PMC5810184; doi:10.1186/s12889-018-5153-y)
Supplement: Supplementary file 1 — Supplementary Materials. Table S1. Marion County population by sex and age. Table S2. Marion County population by race and age. (PDF 43 kb) [file 12889_2018_5153_MOESM1_ESM.pdf]

Supplementary Materials

Table S1. Marion County population by sex and age

**Year**	**Total Residents of Marion County***	**Residents of Marion County by age***			**Female Residents of Marion County***			**Male Residents of Marion County***		
		<65	65-74	>=75	<65	65-74	>=75	<65	65-74	>=75
2007	878,191	784,938	47,621	45,632	404,731	24,554	23,529	380,207	23,067	22,103
2008	883,107	788,646	48,838	45,623	406,567	25,177	23,520	382,079	23,661	22,103
2009	890,878	795,959	49,561	45,358	410,267	25,546	23,379	385,692	24,015	21,979
2010	904,789	808,390	50,842	45,557	418,421	26,316	23,580	389,969	24,526	21,977
2011	911,296	814,130	51,663	45,503	421,428	26,743	23,554	392,702	24,920	21,949
2012**	912,089	812,849	54,137	45,103	420,383	27,998	23,326	392,466	26,139	21,777
2013	913,200	812,045	56,112	45,043	419,625	28,996	23,276	392,420	27,116	21,767
2014	914,475	813,571	56,814	44,090	419,993	29,329	22,761	393,578	27,485	21,329

*Populations are estimates obtained from Marion County Health Department
**Ordinance
Year

Table S2. Marion County population by race and age

Year	Black Residents of Marion County*			White Residents of Marion County*		
	<65	65-74	>=75	<65	65-74	>=75
2007	201,627	12,232	11,721	512,973	31,121	29,821
2008	203,674	12,613	11,782	511,124	31,652	29,568
2009	206,229	12,841	11,752	511,905	31,874	29,171
2010	220,540	13,870	12,429	492,262	30,960	27,742
2011	220,349	13,983	12,316	495,792	31,462	27,711
2012**	224,439	14,948	12,454	486,375	32,393	26,988
2013	226,304	15,637	12,553	480,731	33,218	26,665
2014	228,821	15,979	12,401	476,312	33,262	25,813

*Populations are estimates obtained from Marion County Health Department

**Ordinance Year
